# Supplementary material for: Splice-Junction-Based Mapping of Alternative Isoforms in the Human Proteome
Source: Cell Rep. Author manuscript; Available in PMC 2020 Jan 15. (PMC6961840; doi:10.1016/j.celrep.2019.11.026)
Supplement: 2 [file NIHMS1546469-supplement-2.zip › DF1/PXD010154/Liver/Liver_6_MAP4_MYHDDDLADLVFPSSATADTSIFAGQNDPLKDSYVPLELAK.pdf]

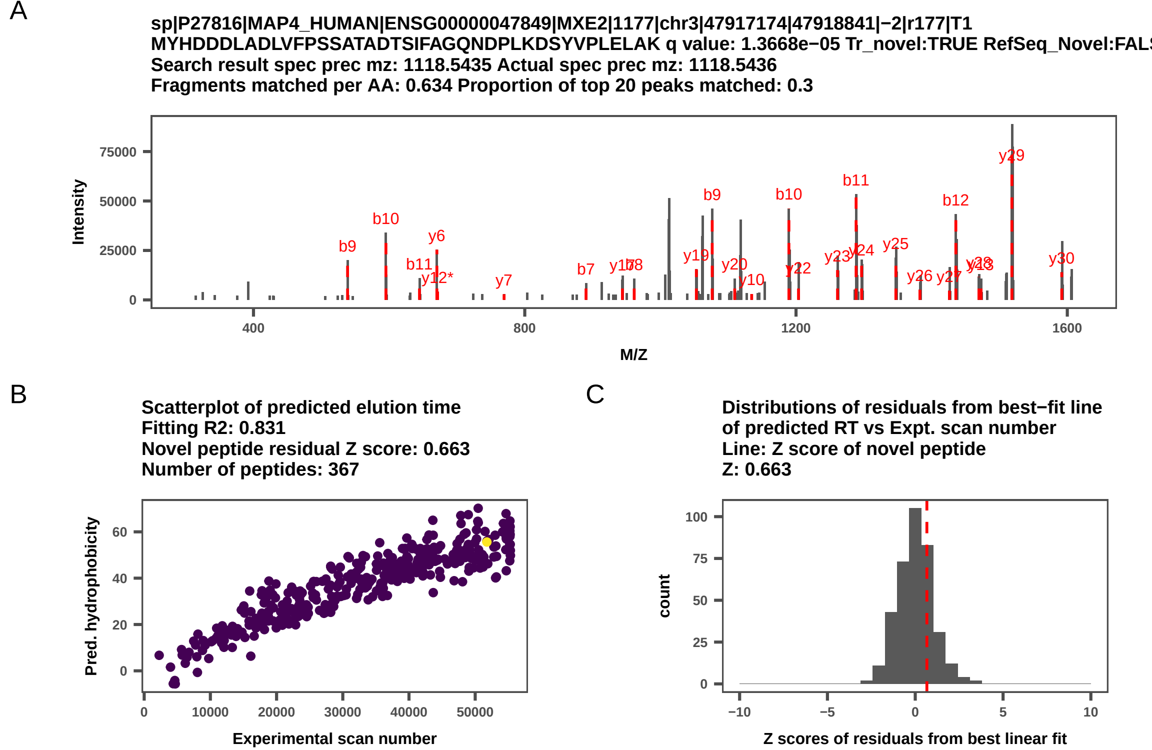
A
sp|P27816|MAP4_HUMAN|ENSG00000047849|MXE2|1177|chr3|47917174|47918841|−2|r177|T1
MYHDDDLADLVFPSSATADTSIFAGQNDPLKDSYVPLELAK q value: 1.3668e−05 Tr_novel:TRUE RefSeq_Novel:FAL
Search result spec prec mz: 1118.5435 Actual spec prec mz: 1118.5436
Fragments matched per AA: 0.634 Proportion of top 20 peaks matched: 0.3
Intensity
75000
50000
25000
0
b9
b10
b11
y6
y12*
y7
b7
y19
b9
y20
y10
b10
y22
b11
y23
y24
y25
y26
y27
b12
y29
y30
400
800
1200
1600
M/Z
B
Scatterplot of predicted elution time
Fitting R2: 0.831
Novel peptide residual Z score: 0.663
Number of peptides: 367
Pred. hydrophobicity
60
40
20
0
0
10000
20000
30000
40000
50000
Experimental scan number
C
Distributions of residuals from best−fit line
of predicted RT vs Expt. scan number
Line: Z score of novel peptide
Z: 0.663
count
100
75
50
25
0
−10
−5
0
5
10
Z scores of residuals from best linear fit
